# Realism in Immune Ecology Studies: Artificial Diet Enhances a Caterpillar's Immune Defense but Does Not Mask the Effects of a Plastic Immune Strategy

**DOI:** 10.3389/finsc.2021.754571

**Published:** 2022-01-21

**Authors:** Eduardo C. Costantin, Daniel L. Viol, Nathalia P. Del Puppo, Simon L. Elliot

**Affiliations:** Laboratory of Insect-Microbe Interactions, Department of Entomology, Universidade Federal de Viçosa, Viçosa, Brazil

**Keywords:** eco-immunology, density-dependent prophylaxis, nutrition, *Anticarsia gemmatalis*, costs of immunity, insect-plant interactions

## Abstract

The immune system is considered a functional trait in life-history theory and its modulation is predicted to be costly and highly dependent on the host's nutrition. Therefore, the nutritional status of an individual has a great impact on an animal's immune ecology. Herbivorous insects are commonly used as model organisms in eco-immunology studies and the use of an artificial diet is the predominant rearing procedure to test them. However, this diet differs from what herbivores experience in nature and it is unclear to what degree this distinction might impact on the relevance of these studies for the real world. Here, we compared plant-based *vs*. artificial diet in a set of three experiments to investigate the interaction of both diets with a plastic immune strategy known as Density-Dependent Prophylaxis (DDP). We used as a model organism the velvetbean caterpillar *Anticarsia gemmatalis*, which is known to adjust its immune defense in line with the DDP hypothesis. Our main results showed that larvae fed with artificial diet had 20.5% more hemocytes circulating in the hemolymph and died 20% more slowly when infected with an obligate (viral) pathogen. Crucially, however, we did not find any indication of fitness costs related to DDP. The use of artificial diet did not interact with that of DDP except in the case of host survival after infection, where the DDP effect was only observable in this diet. Our findings suggest the use of an artificial diet does not mask resource allocation conflicts between immune investment and fitness related traits, but to some extent it might lead to an overestimation of immune parameters and host survival time after infection. We believe that this is the first study to compare an artificial diet and a host plant covering all these aspects: immune parameters, life-history traits, and host survival after infection. Here we provide evidence that, besides the quantitative effects in immune parameters and host survival time, the use of artificial diet interacts only marginally with a density-dependent immune response. This provides support for the use of artificial diets in eco-immunology studies with insects.

## Introduction

Much of the field of animal immune ecology is based on laboratory studies, wherein the investment in immune defense is assessed in response to an artificial challenge (e.g., encapsulation of a nylon fiber) or by challenging the animal with a live pathogen. Also, the studied organisms, commonly insects, are subjected to variations in one or more environmental variables that can shape their immune defense in response to both types of challenges. This approach has been fruitful in showing how diverse environmental variables may affect immune defense, for example the density of conspecifics (1–4), diet (5–8) and temperature (9, 10).

These laboratory studies inevitably simplify the artificial environment for experimental and practical reasons. Thus, temperature may be constant, animals may be alone or in groups of fixed numbers and food may be provided *ad libitum*. Also, the use of an artificial diet is common, especially when it comes to lepidopteran insects [e.g., (11, 12)] which comprise a significant part of such studies. Artificial diets have specifically been developed to favor development of the test animals and their use is justified for standardization of the individual's nutritional status. There are also significant savings in labor, cost and time taken to perform experiments (13).

Where insects are used it has been shown that diet can have profound effects on immune investment. For example, differences in host-plant (14), amount of protein consumption (15) and quality of the protein (6, 16) can modulate an individual's immune status. Further, use of artificial diets facilitates manipulation of specific nutrients to investigate effects on immune defense, applying the Geometric Framework for nutrition using insects as models (7, 17, 18). At some point, however, a question that arises is whether the findings of laboratory studies will translate to the field. Key to this is whether experimental results found with artificial diet will hold when insects are given food that is similar to what they will experience in nature.

We chose to examine this question using the velvetbean caterpillar *Anticarsia gemmatalis* Hübener 1,818 (Lepidoptera:Erebidae). *Anticarsia gemmatalis* investment in immunity is modulated by the conspecific density at which these caterpillars are reared, in line with Density-Dependent Prophylaxis (DDP) hypothesis (19). This hypothesis predicts that organisms can use the perception of increasing population density as an indicator of a greater risk of infection, investing more resources in immune defense in such circumstances. Such a phenotypically plastic strategy to adjust the immune system is probably dependent on the individual's nutritional status, like most immune functions in insects (20). The use of *A. gemmatalis* as a model organism allows us to investigate how the use of an artificial diet interacts with DDP and, consequently, with immune investment, its costs and the course of a pathogenic infection. To our knowledge, this is the first study that aims to investigate not just the quantitative differences in immunity and fitness comparing an artificial diet and a natural diet, but also the interaction with a plastic immune strategy.

Under field conditions, *A. gemmatalis* larvae rely mostly on plants as food for their development. However, host-plants as a source of food do not contain a high-quality protein such as casein (in contrast to most artificial diets); and are usually suboptimal diets for herbivores (21). Plants can also impose a different type of challenge by their secondary metabolites (22). Concentrations of the flavonoids rutin and gestinin in soybean *Glycine max* (L.) Merr. can confer some degree of resistance to herbivory, and consequently impact life-history traits (23). This set of characteristics is completely distinct from what those larvae experience in a set of laboratory experiments using artificial diet.

When plant-based and artificial diets have been compared, larval development, pupal weight and female fecundity were enhanced in lepidopteran species on artificial diet (24–26). These results represent an impact on life-history traits and fitness. Concerning immune function, assuming that this trait also demands energy and protein from food (27), the nutritional differences of artificial diet and plants will probably impact immune investment in these larvae. Moreover, since immunity is costly (28–31) it is possible that we can also find different patterns in how the costs of prophylaxis are translated to fitness comparing both diets. If the different biological systems of an organism (e.g., immune and reproductive system) compete internally for the same resources, the resource allocation to one of them could constrain the other (31). In terms of prophylactic defense against parasites, the different quality of food ingested can circumvent allocation trade-offs between immunity and other life-history traits (32). As a result, the use of artificial diet to investigate the costs of immune investment can represent an unrealistic situation that masks resource allocation conflicts.

The differences between an artificial diet and plant leaves lead us to question if we are not losing information regarding immune defenses and their interaction with other fitness related traits, such as development times and reproductive output. Thus, we aim here to understand the consequences of using artificial diet in immune-ecology studies. We performed three experiments using density (1–4 larvae/per pot) and diet (artificial diet or soybean leaves) as treatments to investigate: (i) the differences in immune parameters (ii) the life-history traits and the costs related to the prophylactic investment in immunity and (iii) the susceptibility to an obligate-killing pathogen *Baculovirus anticarsia* (AgMNPV) in *A. gemmatalis*. We hypothesized that the artificial diet alters the effect of density-dependent prophylaxis, more specifically we predict that crowded larvae will be able to mount an immune response with reduced fitness costs when fed with this diet.

## Materials and Methods

### Insect Colony

The *Anticarsia gemmatalis* colony was established in the Laboratory of Insect-Microbe Interactions at the Universidade Federal de Viçosa in 2013 with insects from the Laboratory of Biological Control at EMBRAPA/CNPMS. Embrapa's colony was supplemented periodically with wild insects and we added new individuals from a stock colony (EMBRAPA/CNPSO) once. These insects are kept in the laboratory under controlled conditions of 25 ± 2°C, 65 ± 10% relative humidity and 12:12 h light cycle. The moths were housed in groups of ca. 80 pairs in wooden cages (measuring 30 × 30 × 30 cm) lined with paper sheets (which served as an oviposition substrate) and fed *ad libitum* with a liquid diet consisting of water, honey, beer, saccharose, ascorbic acid and nipagin—methodology adapted from Hoffmann-Campo et al. (33). The eggs were collected every 72 h and kept in plastic pots (1 liter) containing plugs of artificial diet until they hatched. Upon hatching, larvae were transferred to plastic pots (100 ml) at densities of 1–4 individuals per pot. These densities are known to trigger color phenotypic changes (12). They were then used to maintain the expression of the solitary (1 individual) and the high-density phenotype (4 individuals).

### Diet - Artificial Diet and Soybean Leaves

The artificial diet used to feed the larvae to maintain the colony was the same used to perform the experiments. It is composed of textured soy protein, bean, wheat germ, beer yeast, nipagin, ascorbic acid, sorbic acid, formaldehyde, agar-agar, casein and vitamin solution [adapted from (33)]. Casein is a high-quality protein, thus we consider the artificial diet as a high-quality diet. Soybean [*Glycine max* (L.) Merr.] plants used in this study were of the variety “Williams 82.” The seeds were obtained from the Laboratory of Genetic and Genomic Plant-Pathogen Interactions, Universidade Federal de Viçosa (LGGIP-UFV). Sowing occurred weekly in 500 ml plastic cups containing a mixture of plant substrate composed of *Pinus* bark, peat and vermiculite (Tropstrato HT Hortaliças), sand and soil. The cups were placed in cages covered with gauze to avoid prior contact with other herbivores. Plants were provided with macronutrients one week after seed germination following recommendations from Sfredo (34). The cages were placed outside and plants were watered once a day. The leaves used in the experiment were fully developed leaves, with no symptoms of disease and were obtained from the plants before they reached the R4 stage (full pod).

### Experimental Design and Color Phenotypes

We conducted three experiments with the same experimental design, in which the independent variables were larval density and diet, as described below. They were conducted one after the other for logistical reasons. In two of the three experiments, data on larval color phenotypes were collected. This variable allowed us to check the experiments for consistency and also to determine that the density treatment was having its expected effect on phenotype (see below).

The first experiment was conducted to perform immune assays, the second to collect data on life-history traits and the third to check larval susceptibility to *baculovirus anticarsia* (AgMNPV). Newly emerged larvae were placed in 100 ml pots and assigned to a 2 × 2 factorial design, completely randomized, as follows: density (1 or 4 larvae per pot) and diet (artificial or soybean leaves). It has been shown for this species that the presence of just 1 conspecific is sufficient to trigger changes in larvae phenotype (12). From a practical point of view, we chose the density of 4 larvae in the high density treatment as it made it relatively easy to maintain hygiene in the recipient until the end of the experiment (with more than 4 larvae/pot the proliferation of bacteria and fungi is high).

While the artificial diet was added only at the beginning of the experiment, the leaflets had their petioles wrapped in damp cotton and were replaced as necessary—pots were checked twice daily and, ~1 leaflet was replaced for solitary larvae and two leaflets for high density larvae per day. With respect to the high density treatment, we randomly selected the individual (by numbering them and a second person choosing a number between 1 and 4) to perform the experiments on the tenth day (experiment I and II) or on the eight day (experiment III). To simulate similar manipulation conditions, pots containing artificial diet were also manipulated at the same frequency as the pots containing leaves.

The phenotypic classification was done in two experiments on the tenth day after egg eclosion. As described by Fescemyer and Hammond (35) there are visual differences between the phenotypes, especially body and head capsule coloration. Pictures of the different phenotypes can be found in Silva et al. (12). To classify the larvae, we visually observed the olive-green and the black discriminatory coloration first, then the individuals that did not fit in one of these categories were classified as intermediate. This visual classification was used (rather than using digitalized images) as we wished to avoid overmanipulating the larvae. Manipulation could affect development and trigger changes in the phenotype (D.L.V. *personal observation*).

### Experiment I - Immune Assays

We started the experiment with 45 replications per treatment, larvae that did not survive until the tenth day were excluded. On the tenth day after eclosion, caterpillars of the immunoassay test group were weighed and two immune parameters were assessed.

#### Hemocyte Numbers

A sample of 2·5 μl of hemolymph was collected from each individual by puncturing a small hole beside the first prolegs using a thin needle. This was added to an Eppendorf tube with 20 μl of anticoagulant buffer (98 mM NaOH, 186 mM NaCl, 17 mM NaEDTA and 41 mM citric acid, pH 4.5). Two aliquots of 10 μl of the suspension were added to each side of a Neubauer improved chamber and total hemocytes were counted under a microscope (Nikon eclipse E200). The final value was the mean of the two aliquots multiplied by the Neubauer chamber correction factor, providing the numbers of hemocytes per microliter [adapted from (36)].

#### Lytic Activity

Lysozyme antibacterial activity was measured through the inhibition zones formed in agar plates containing the bacterium *Micrococcus lysodeikticus* ATCC 4968 (37). Plates were prepared with 1.5% agar, 0.75 g of *M. lysodeikticus* in lyophilized form, 100 ml distilled water and 50 ml of 2M potassium phosphate buffer 2M (pH 6.4). Holes were punched in the agar after it had hardened with the aid of glass capillary tubes (1.5 mm diameter). Two aliquots of 1 μl of hemolymph per sample were pipetted into the holes of agar plates. Plates were incubated at 33°C for 24 h and then photographed. The areas of clear zones formed in the vicinity of the holes were measured with the aid of ImageJ software (1.51w version) and were transformed to diameters (mm).

### Experiment II – Life-History Traits

We started the experiment with 70 replications per treatment, then the larvae were randomly divided after pupation into 2 groups: one to assess longevity and lifetime potential fecundity and the other one to estimate fat content. To assess the costs of immune defenses in *A. gemmatalis*, we assessed the following life-history parameters:

#### Larval and Pupal Development and Pupal Weight

To characterize larval and pupal development we observed: (i) caterpillar (egg to pupa) and (ii) pupal development times. For this, pots were checked every day. Pupation was characterized by the larvae changing to a brown coloration and assuming a barrel shape. Pupation ended when adults emerged. The individuals were sexed using a stereomicroscope (Olympus SZ61) based on the sexual dimorphism presented in the pupal phase, according to Hoffman-Campo et al. (33). Pupal weight was also obtained from each individual on the second day post pupation, using an analytical balance (Bel Engineering - Mark M. Osasco SP/Brazil).

#### Longevity and Lifetime Potential Fecundity

Half of the newly emerged adults from the life-history experiment were placed in 300 ml pots with a falcon tube cap containing a piece of cotton moistened with the same liquid diet used to maintain the laboratory colony (see Insect Colony). The diet was replaced every two days and the moths could feed freely. A piece of gauze was used to cover the pot, thus avoiding escape and excessive humidity. To determine adult longevity, the number of dead adults in each of the treatment groups was counted and recorded daily until all adults died. The moths were considered dead when they seemed to be immobile in the pot and did not move even after being touched with tweezers. Lifetime potential fecundity was determined by recording the number of eggs laid in the pots plus the eggs found remaining in the oviducts upon dissection. Since mating in this species seems to occur only under grouping conditions (E.C.C. *personal observation*) fecundity was estimated from virgin females. Lifetime potential fecundity of virgin females could be indicative of female reproductive potential. This is based upon Kemp and Rutowski (38, 39) in other species of Lepidoptera.

#### Fat Content

The remaining newly emerged moths from the life-history experiment were used to estimate the fat content. We dried males and females on the day of emergence in a drying oven (Ethik Technology - 402 D. Vargem Grande Paulista SP/Brazil) at 60°C for 48 h. After this drying period, we weighed the thorax and abdomen using a Shimadzu ATX 224 microbalance (precision 0.1 mg) and placed the body parts in a microtube containing 3 ml chloroform for 48 h—chloroform is a solvent used to extract only lipids. Then we returned the fatless body parts to a drying oven for an additional 24 h and reweighed them [methodology adapted from (40, 41)]. Drying oven and chloroform time periods were adjusted based on previous tests. We calculated fat content as the percentage of fat extracted in chloroform in relation to the moth thorax and abdomen initial weight.

### Experiment III – Susceptibility to *Baculovirus anticarsia* (AgMNPV)

The virus used in this experiment was provided by CNPSo-EMBRAPA. We obtained the viral suspension from dead caterpillars (kept frozen at −20°C) which were inoculated previously with AgMNPV and showed clear signs of the virus infection. Eight days after the beginning of the experiment, the larvae from each density × diet treatment were separated individually and kept without diet for 24 h. We offered, for additional 24 h, a square soybean leaf piece inoculated with 10 μl of virus suspension (1 × 10^6^ polyhedra/larvae) plus Tween 80 (0.01%). After this period, we returned the larvae to their original diet treatment. Larvae that did not consume the entire leaf piece in this period were excluded from the experiment. In the control group, larvae were fed with soybean leaf pieces inoculated with 10 μl of distilled water plus Tween 80. Mortality was assessed daily until death or pupation. Larvae were considered dead by AgMNPV if they did not react to mechanical stimulation (see above) and had clear symptoms of virus infection: they become pale and flaccid and they release a thick white fluid after the cuticle ruptures.

### Statistical Analyses

The effects of density and diet (variables used in the model) on immunity and life-history traits of *A. gemmatalis* were verified using Generalized Linear Models (GLM) followed by an analysis of variance (ANOVA). The analyses were carried out by fitting a full model and then simplifying it by excluding the non-significant interaction and terms. The final model was accepted as the simplest model that was not significantly different from the full model. For data that did not fit in the normal distribution and were overdispersed (hemocyte numbers), the negative binomial distribution with log-link function was used. When data fit the normal distribution, the Gaussian family was used (lytic activity, number of eggs, fat content and pupal weight). The models were compared using an *F*-test (*p* > 0.05). A survival analysis with a Weibull distribution was conducted for parameters such as development time of larvae, pupal period, longevity and survival after baculovirus inoculation. For model comparison in the survival analysis we used the χ^2^ test (*p* > 0.05). Results were presented as means ± SE. All analysis was done using the software R (version 3.4.1).

## Results

### Phenotype Frequencies

**Figure 1:**
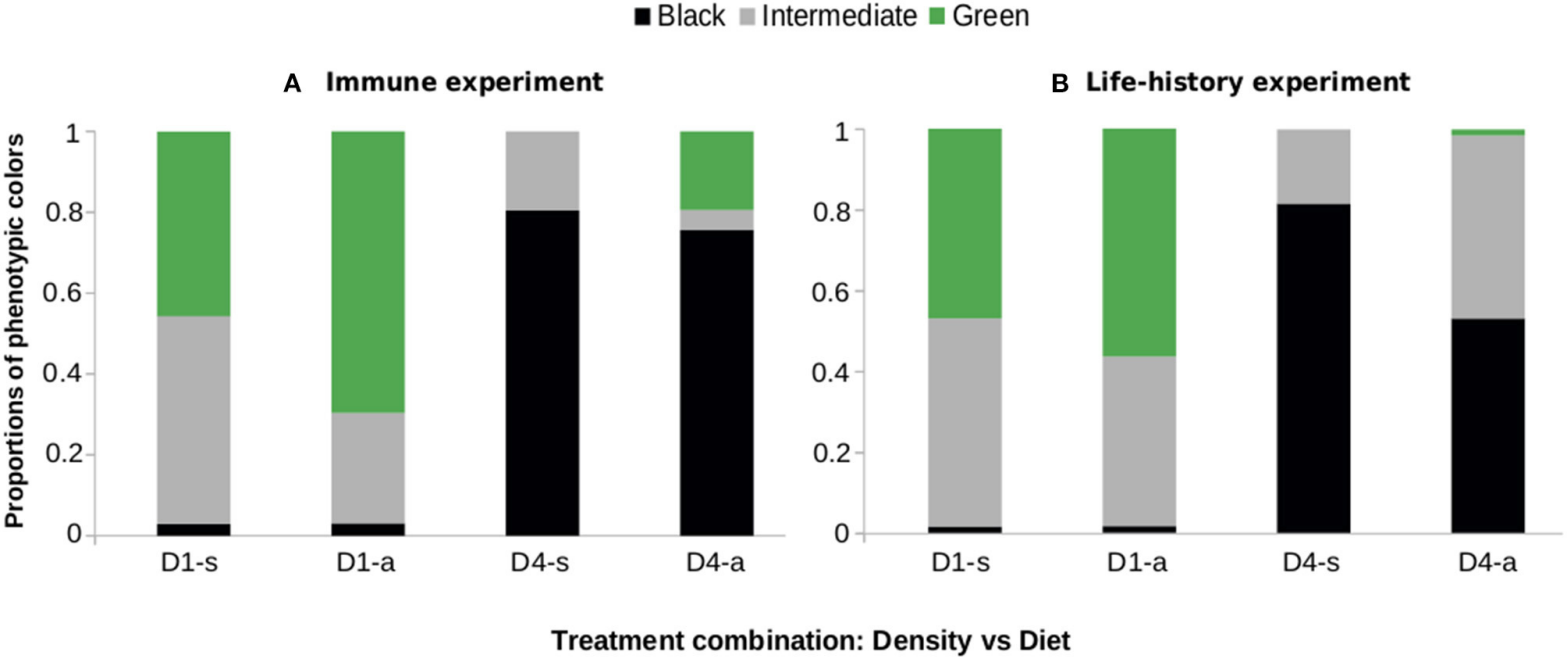
Proportions of green, intermediate and black phenotypes in caterpillars of *Anticarsia gemmatalis* as a function of density (1–4 larvae/per pot, “D1” or “D4” respectively) and diet (artificial diet “a” or soybean leaves “s”). **(A)** Experiment I and **(B)** Experiment II (see text for details). The color of each caterpillar was defined visually on the tenth day after egg eclosion.

To check the similarities between the experiments, we assessed phenotype frequencies in two of them. *Anticarsia gemmatalis* expressed different color phenotypes—black, intermediate or green, according to the rearing density: 1–4 larvae per pot (Figure 1). In both experiments, when reared alone (D1) the green phenotype was prevalent—over 45%, while the black phenotype was the least expressed. Meanwhile, at high densities (D4) the black phenotype was predominant—over 57%—and the green phenotype the least expressed.

### Experiment I – Immune Assays

#### Hemocyte Number

**Figure 2:**
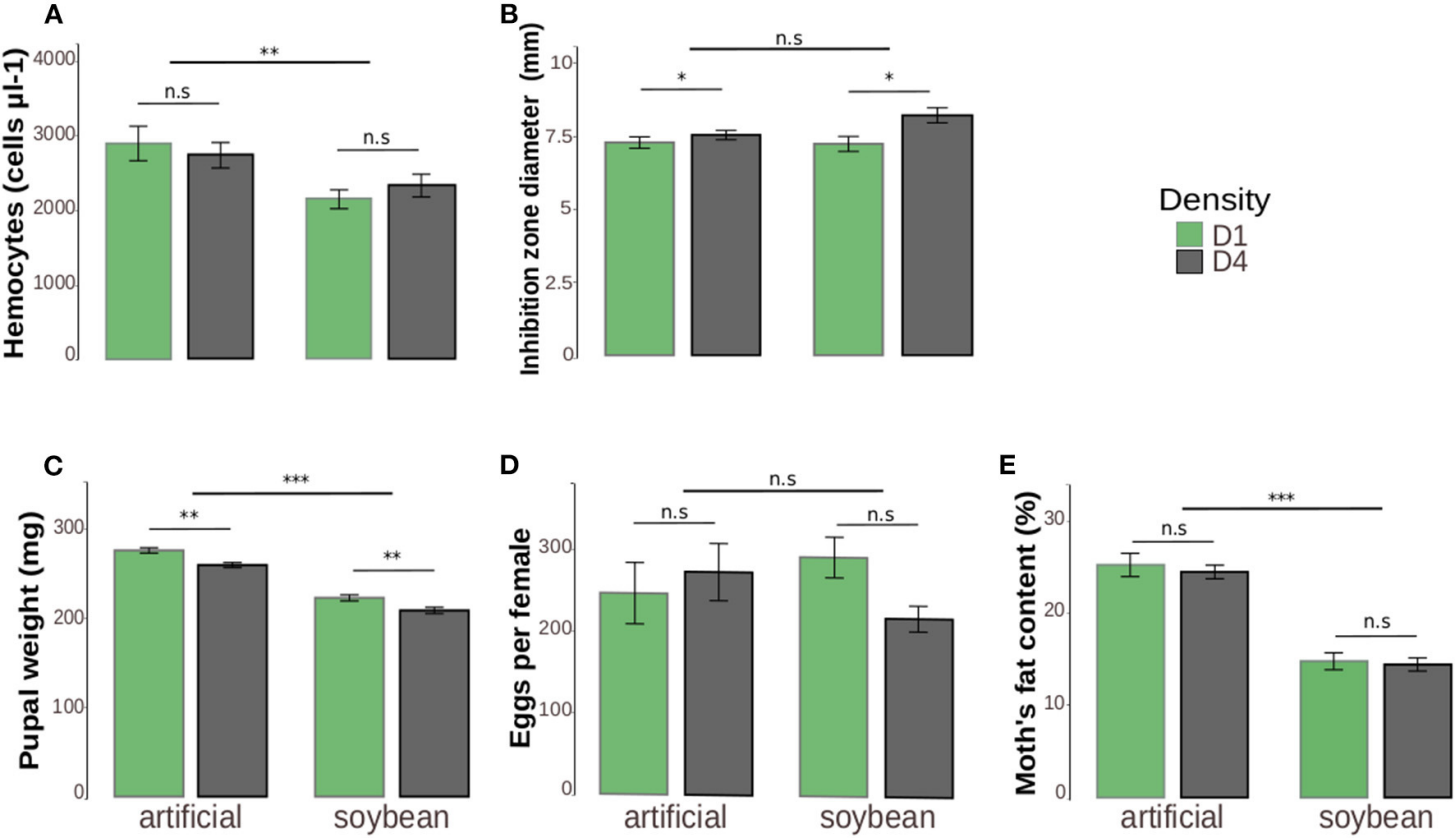
Immune parameters **(A,B)** and life-history traits **(C,E)** of *Anticarsia gemmatalis* as a function of density (1–4 larvae/per pot: “D1” or “D4” respectively) and diet (artificial diet or soybean leaves). The results are represented by the means ± standard error (SE). **(A)** Hemocyte number (cells μl-1) in the hemolymph. The total hemocyte number was counted in a Neubauer improved chamber under a microscope. **(B)** Diameter of inhibition zone area (mm) formed by the lysozyme activity from hemolymph. Two aliquots of 1 μl of hemolymph per sample were pipetted into the holes of agar plates containing the bacterium *Micrococcus lysodeikticus*. The areas of clear zones formed in the vicinity of the holes were measured with the aid of ImageJ software and transformed to diameters. **(C)** Pupal weight (mg). The pupal weight from each individual was obtained on the second day after they had pupate. **(D)** Lifetime potential fecundity represented by the number of eggs per female. Fecundity was determined by recording the number of eggs laid in the pot plus the number of eggs remaining in the female oviducts on the day of death. **(E)** Percentage of fat content. The fat content was calculated as the percentage of fat extracted in chloroform in relation to the moth thorax and abdomen initial weight. *p ≤ 0.05; **p ≤ 0.01; ***p ≤ 0.001.

The mean value of circulating hemocytes in hemolymph was 20.5% lower in larvae fed with soybean leaves (2.232 ± 139.1 cells μl-1) in comparison to artificial diet (2.808 ± 200.7 cells μl-1) [*Z*_(1, 150)_ = −3.526, *p* = 0.0004). Density [*Z*_(1, 148)_ = −0.617, *p* = 0.8202] and the interaction between density and diet [*Z*_(1, 149)_ = 1.085, *p* = 0.2778] were not significant (Figure 2A).

#### Lytic Activity

The conspecific density affected positively the diameter of inhibition zones. For solitary larvae (D1) the mean diameter was 7.265 ± 0.22 mm, while for high density (D4) individuals, was 7.883 ± 0.20 mm [*F*_(1, 150)_ = 7.54, *p* = 0.0057]. There were no significant differences in relation to larval diet [*F*_(1, 151)_ = 2.36, *p* = 0.1217] or interaction between the two variables [*F*_(1, 149)_ = 2.44, *p* = 0.119] (Figure 2B).

### Experiment II - Life-History Traits

#### Development Times - Larvae and Pupae

Larval development times did not vary with diet [$\chi_{\left(1\right)}^{2}$ = 4.00, *p* = 0.0504] or density [$\chi_{\left(2\right)}^{2}$ = 1.31, *p* = 0.2921]. Pupal times, though, did vary with diet [$\chi_{\left(2\right)}^{2}$ = 69.93, *p* < 0.0001], with larvae reared on artificial diet remaining longer as pupae (10.4 ± 0.584 days) than those reared on soybean leaves (9.72 ± 0.605 days) (Table 1).

**Table 1:** Duration [days ± standard error (SE)] of *Anticarsia gemmatalis* development stages (larval period, pupal period and moth longevity) as a function of density (1 or 4 larvae/per pot: “D1” or “D4” respectively) and diet (artificial diet “a” or soybean leaves “s”).

**Treatment combination**	**Period (days** **±SE)**					
	**Larval**	***N***	**Pupal**	***N***	**Longevity**	***N***
D1-s	13.19 ± 0.094	66	09.71 ± 0.074	66	18.44 ± 0.964	32
D1-a	13.40 ± 0.092	65	10.49 ± 0.079	65	18.81 ± 0.979	31
D4-s	13.19 ± 0.107	67	09.73 ± 0.073	67	18.00 ± 0.934	33
D4-a	13.35 ± 0.090	69	10.31 ± 0.064	69	19.81 ± 0.988	32

#### Longevity of Moths

The longevity of moths was not affected by diet [$\chi_{\left(1\right)}^{2}$ = 1.31, *p* = 0.252] or density [$\chi_{\left(2\right)}^{2}$ = 0.02, *p* = 0.0941] (Table 1).

#### Pupal Weight

Pupae fed with artificial diet as larvae were 20% heavier than those fed with soybean leaves [*F*_(1, 264)_ = 259.87, *p* < 0.0001]. With respect to density, pupae reared alone (D1) were ~6% heavier than those reared at high densities (D4) [*F*_(1, 265)_ = 22.83, *p* < 0.0001]. However, there was no interaction between the two factors [*F*_(1, 263)_ = 0.118, *p* = 0.730] (Figure 2C).

#### Lifetime Potential Fecundity

The potential fecundity of the females did not differ in relation to diet [*F*_(1, 51)_ = 0.005, *p* = 0.943] or density [F_(1, 52)_ = 0.288, *p* = 0.590]. Also, no interaction between diet and density was found [*F*_(1, 50)_ = 2.49, *p* = 0.120] (Figure 2D).

#### Fat Content

Moths that were fed with artificial diet as larvae had more fat in their bodies (24.8%) than those reared on soybean (14.6%) [*F*_(1, 125)_ = 144.50, *p* < 0.0001]. Density [*F*_(1, 126)_ = 0.049, *p* = 0.824] and the interaction [*F*_(1, 124)_ = 0.044, *p* = 0.8243] were not significant (Figure 2E).

### Experiment III - Susceptibility to *Baculovirus anticarsia* (AgMNPV)

**Figure 3:**
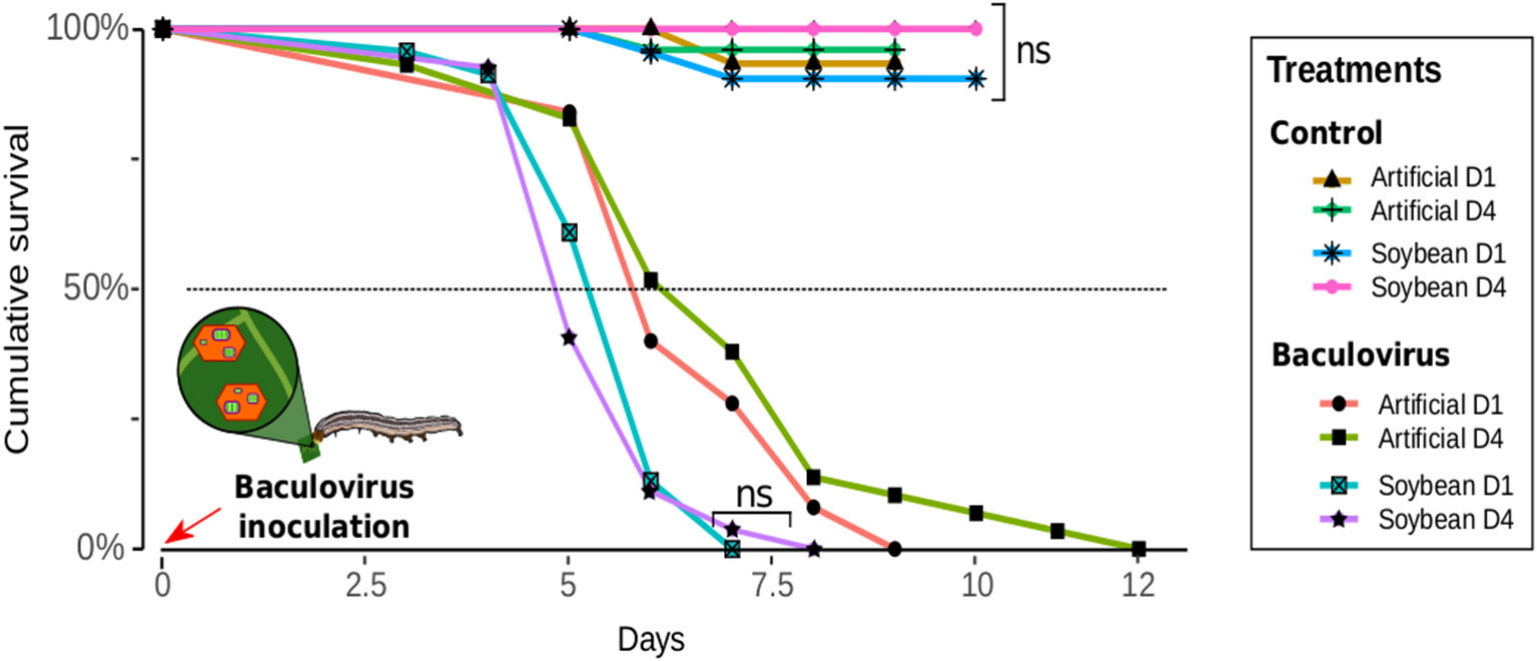
Susceptibility of *Anticarsia gemmatalis* to *Baculovirus anticarsia* (AgMNPV) as a function of density (1–4 larvae/per pot: “D1” or “D4” respectively) and diet (artificial diet or soybean leaves). Insects of the Baculovirus treatment group were inoculated with 10 μl of suspension (1 × 10^6^ polyhedra/larvae) plus Tween 80 (0.01%), while those of the control treatment were inoculated with 10 μl of distilled water plus Tween 80 (0.01%). Larval mortality or pupation were assessed daily.

Larvae fed with soybean leaves died 20% faster after infection compared with the ones fed with artificial diet [$\chi_{\left(6\right)}^{2}$ = 181.12, *p* < 0.001]. Meanwhile, the effect of density was only visible when the artificial diet was provided [D4-soybean vs. D1-soybean, $\chi_{\left(6\right)}^{2}$ = 192.47 *p* = 0.857; D1-artificial vs. D4-artificial, $\chi_{\left(2\right)}^{2}$ = 187.37, *p* = 0.020]. The mean survival times after a pathogenic challenge of larvae fed with soybean leaves were 5.60 ± 0.19 and 5.48 ± 0.17 days; while those for larvae on artificial diet were 6.60 ± 0.24 days and 6.93 ± 0.37 days (D1 and D4, respectively) (Figure 3).

## Discussion

Artificial diets have been useful to rear and study lepidopteran species in laboratory conditions, but their effects on immune ecology have not been investigated to date. Using a phase-polyphenic caterpillar, our study aimed to understand the consequences of using an artificial diet in an eco-immunology context. Overall, we demonstrate that the use of an artificial diet does not significantly interfere in density-dependent prophylaxis (DDP), but may enhance immune defense and larval performance after a pathogenic challenge. We expected that the addition of soybean leaves—a natural food—could reveal negative interactions between the immune system and other fitness traits in crowded larvae (D4), but this was not the case in our system.

In line with the DDP hypothesis and previous empirical studies on this caterpillar (12), the higher density treatment had its expected effect on *A. gemmatalis* since we observed different phenotypes in crowded and solitary larvae (Figure 1). Given this, we assessed immune defense directly through the hemocyte number and the diameter of inhibition zones due to antimicrobial protein, lysozyme. The hemocyte number is known to vary according to diet (42, 43) and *A. gemmatalis* larvae fed with artificial diet had higher hemocyte densities (Figure 2A). Producing and maintaining higher numbers of hemocytes in the hemolymph is energetically demanding (44, 45), so it is expected that the individuals that have access to more balanced food can sustain this cellular defense at higher levels. Lysozyme production has presented contradictory results in species that manifest DDP (2, 11), possibly due to a trade-off between lysozyme and phenoloxidase activity (46). Here, lysozyme activity was positively affected by the presence of conspecifics (Figure 2B), which is an expected outcome in the context of DDP. Regarding immune defense, the artificial diet did not change the pattern found on soybean leaves in solitary and crowded larvae.

To look for possible costs of DDP we measured larval, pupal and adult life-history traits. Negative impacts on fitness related traits such as pupal mass, fat content, fecundity or longevity in high density larvae could be an indication of DDP costs. Pupal mass was dependent on both density and diet and lower pupal masses were observed in crowded larvae on both diets (Figure 2C). Fescemyer and Erlandson (47) found similar results in the same species. In the immune system assessment, crowded larvae had a slightly higher production of lysozyme, but we do not believe that this could be responsible for a reduced pupal weight in this treatment group.It is not clear if the reduced weight was due to the investment in other immune responses, such as phenoloxidase activity (48), or larval competition in the earlier instars. Nevertheless, greater pupal weight is a good predictor for fitness in insects (49), so we expected to find considerable differences in fitness traits (e.g., fecundity and longevity).

Larvae reared on artificial diet became adults with a higher percentage of fat in their bodies (Figure 2E). Fat content plays an important role in an insect's life-history and can influence adult longevity and female fecundity (50). In a pilot experiment with no carbohydrate offered to adults we observed that all the moths from the soybean leaf treatment died by the third day after pupae emergence, indicating limited nutritional reserves (51), while those from the artificial diet all remained alive (E.C.C *personal observation*). These results and our observation is an indication that fat content is important for, at least, adult longevity in *A. gemmatalis*.

Contrary to our expectations, neither longevity nor fecundity of adults was affected by larval diet and density (Figure 2D; Table 1). The quality of food that holometabolous insects ingest in their immature stages impacts adult longevity (52, 53) and female fecundity (49, 54). However, this does not appear to be true for *A. gemmatalis* when artificial diet and soybean leaves were compared and adults were offered a source of carbohydrate *ad libitum*. We suspect that individuals reared on soybean leaves can compensate the reduced energy reserves acquired in the larval phase (fat content) by compensatory feeding in the adult stage, as has been described in other holometabolous insects (55, 56). This applies in a laboratory setting with food offered *ad libitum*, apparently not in the same setting when offered no food and in the field, where the insects must expend energy and run risks of predation to ingest carbohydrates. We may expect some middle ground where the potential for compensatory feeding is limited but not impossible. Regarding density, the lack of fitness costs evident in either longevity or fecundity indicates there may be no cost of density-dependent prophylaxis in our system. However, we cannot discard the possibility that compensatory feeding behavior could also have masked the costs of DDP in both adult traits.

When challenged with a baculovirus larvae fed with artificial diet had greater survival times than the ones fed on soybean leaves (Figure 3). The higher number of hemocytes of these individuals can explain this outcome, since hemocytes are an important antivirus defense for insects, mainly due to phagocytosis (57). Hemocytes play a central role in host resistance in biological systems involving baculovirus and lepidoptera (58, 59). Interestingly, the density effect predicted by the DPP hypothesis was marginally observed when the artificial diet was consumed. However, despite the interaction with the DDP, the artificial diet is not masking any effect of a plant-based diet.

There was no indication of physiological life-history costs related to DDP in our system, similar results were found for *Spodoptera litorallis* by Cotter et al. (60). Host-plants are commonly considered suboptimal food for insects, especially due to nitrogen limitation (61). If there was any fitness cost related to prophylaxis, it is more likely to appear in those individuals fed on plant material. None of the fitness-related traits (longevity and fecundity) were affected by the density treatment in *A.gemmatalis*, even when the larvae were fed with soybean leaves. Thus, the use of artificial diet did not mask resource allocation conflicts between the immune system and other fitness traits in our study. However, immune function and survival assays using pathogens could differ when a plant or artificial diet were used.

Our initial hypothesis on the costs of using leaves as food on prophylactic immunity was not supported. Nevertheless, the costs of mounting an immune response are condition-dependent and can be revealed with food restriction in larval or adult stages, which is probably a common situation in nature in contrast to provision of food *ad libitum* (62, 63). It may be that food restriction can be sufficient to reveal the costs of DDP.

To our knowledge, our study is the first to compare an artificial diet with natural food in an eco-immunology context. This is an important step in a bottom-up process of taking lab-based and often artificial studies in to the field context. We argue that despite the change in immune defense and disease progress, an artificial diet is suitable to perform eco-immunology studies, at least in the context of the DDP hypothesis. Nevertheless, more research is needed to better understand how it will occur with other phenomena such as immune priming. We also encourage the integration of ecological variables (e.g., density of conspecifics, diet, food restriction) to reveal the complex interaction between immunity and other traits.

## Data Availability Statement

The raw data supporting the conclusions of this article will be made available by the authors, without undue reservation.

## Author Contributions

DV, SE, and EC conceptualized the study and wrote the manuscript. EC, DV, ND, and SE designed the experiments. EC, DV, and ND ran the experiments. EC and DV analyzed the results. All authors contributed to the article and approved the submitted version.

## Funding

The authors thank Coordenação de Aperfeiçoamento de Pessoal de Nível Superior (CAPES) – Finance Code 001 - and FAPEMIG (Fundação de Amparo à Pesquisa do Estado de Minas Gerais), - project no. APQ-00123-18 - for providing financial support. SE was in receipt of a CNPq productivity grant no. 309845/2016-5.

## Conflict of Interest

The authors declare that the research was conducted in the absence of any commercial or financial relationships that could be construed as a potential conflict of interest.

## Publisher's Note

All claims expressed in this article are solely those of the authors and do not necessarily represent those of their affiliated organizations, or those of the publisher, the editors and the reviewers. Any product that may be evaluated in this article, or claim that may be made by its manufacturer, is not guaranteed or endorsed by the publisher.
